# Transport Proteins Enabling Plant Photorespiratory Metabolism

**DOI:** 10.3390/plants10050880

**Published:** 2021-04-27

**Authors:** Franziska Kuhnert, Urte Schlüter, Nicole Linka, Marion Eisenhut

**Affiliations:** Institute of Plant Biochemistry, Cluster of Excellence on Plant Science (CEPLAS), Heinrich Heine University Düsseldorf, Universitätsstrasse 1, 40225 Düsseldorf, Germany; franziska.kuhnert@hhu.de (F.K.); u.schlueter@hhu.de (U.S.); nicole.linka@hhu.de (N.L.)

**Keywords:** photorespiration, photosynthesis, transport protein, plant, Rubisco, metabolite, synthetic bypass, C_4_ photosynthesis

## Abstract

Photorespiration (PR) is a metabolic repair pathway that acts in oxygenic photosynthetic organisms to degrade a toxic product of oxygen fixation generated by the enzyme ribulose 1,5-bisphosphate carboxylase/oxygenase. Within the metabolic pathway, energy is consumed and carbon dioxide released. Consequently, PR is seen as a wasteful process making it a promising target for engineering to enhance plant productivity. Transport and channel proteins connect the organelles accomplishing the PR pathway—chloroplast, peroxisome, and mitochondrion—and thus enable efficient flux of PR metabolites. Although the pathway and the enzymes catalyzing the biochemical reactions have been the focus of research for the last several decades, the knowledge about transport proteins involved in PR is still limited. This review presents a timely state of knowledge with regard to metabolite channeling in PR and the participating proteins. The significance of transporters for implementation of synthetic bypasses to PR is highlighted. As an excursion, the physiological contribution of transport proteins that are involved in C_4_ metabolism is discussed.

## 1. Introduction—The Photorespiratory Metabolism

**Figure 1:**
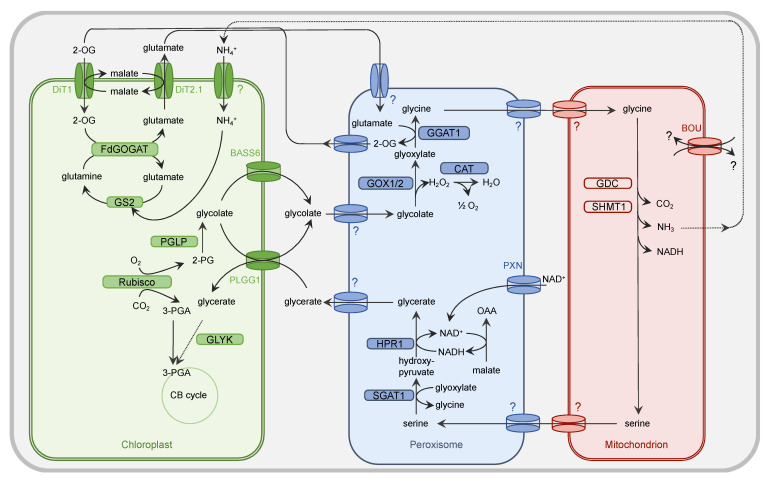
Simplified presentation of the plant PR metabolism. Oxygenation activity of Rubisco within the chloroplast results in the generation of one molecule of 3-PGA and 2-PG each. While 3-PGA is metabolized in the CB cycle, 2-PG is degraded by the PR metabolism. Within the chloroplast, 2-PG is dephosphorylated by 2-PG phosphatase (PGLP). Formed glycolate is exported from the chloroplast by PLGG1 and BASS6 and enters the peroxisome by a yet unidentified (?) translocator. Glycolate oxidase (GOX1/2) generates glyoxylate and H_2_O_2_. The latter one is converted by catalase (CAT) activity into O_2_ and H_2_O. The PR intermediate glyoxylate is aminated by glutamate:glyoxylate aminotransferase (GGAT1) to yield glycine. Glutamate serves as a donor for the amino group. The peroxisomal importer for this amino acid but also glycine is to date unknown. Glycine needs to be imported by an unknown translocator into the mitochondrion. Here, the concerted action of the multienzyme system glycine decarboxylase (GDC) and serine hydroxymethyltransferase (SHMT) results in release of CO_2_ and NH_3_ from one molecule of glycine but also conversion of another molecule of glycine into serine. The substrate(s) of the transporter À BOU DE SOUFFLE (BOU) are unclear. BOU is likely involved in mitochondrial glutamate transport and functionally linked with glycine-to-serine conversion. Export of serine from the mitochondrion is facilitated by an unknown transporter protein. This applies also to the import into the peroxisome. Serine is deaminated by serine:glyoxylate aminotransferase (SGAT1) to form hydroxypyruvate. For the subsequent reduction of this intermediate to glycerate, hydroxypyruvate reductase 1 (HPR1) depends on NADH, which is provided by the oxidation of OAA into malate. NAD^+^ is basically imported into peroxisome by the peroxisomal NAD carrier PXN. Glycerate leaves the peroxisome by an unknown mechanism and imported into the chloroplast by PLGG1. The concluding step in the PR pathway is the phosphorylation of glycerate and formation of 3-PGA by glycerate kinase (GLYK). 3-PGA enters the CB cycle. The dicarboxylate translocators DiT1 and Dit2.1 function as two-translocator system for the refixation of NH_4_^+^ in the chloroplast by the GS/FdGOGAT system. Further details are given in the main body.

Oxygenic photosynthesis builds the foundation of life on earth. By the action of the photosynthesis apparatus, energy from the sun is harvested and converted into chemical energy, with oxygen (O_2_) as a byproduct. The chemical energy drives the Calvin–Benson cycle to fix atmospheric carbon dioxide (CO_2_) and produce sugars. Central to CO_2_ fixation is the enzyme ribulose 1,5-bisphosphate carboxylase/oxygenase (Rubisco). This world’s most abundant protein accomplishes assimilation of ca. 250 billion tons of CO_2_ into biomass per year [1]. As a biochemical reaction, Rubisco catalyzes the condensation of one molecule of ribulose 1,5-bisphosphate with one molecule of CO_2_ to produce two molecules of 3-phosphoglycerate (3-PGA). However, the enzyme also accepts O_2_ as a substrate. In the case of oxygenation activity, Rubisco forms one molecule of 3-PGA and one molecule of 2-phosphoglycolate (2-PG) [2]. 2-PG acts as an inhibitor of enzymes of the central carbon metabolism. These are phosphofructokinase [3], sedoheptulose 1,7-bisphosphatase [4], and triose phosphate isomerase [5]. Confronted with the challenge to perform oxygenic photosynthesis and to thrive, cyanobacteria have evolved the photorespiratory (PR) metabolism to degrade 2-PG rapidly [6,7]. Oxygenic photosynthetic eukaryotes, including algae and land plants, inherited the indispensable ability to perform PR metabolism (reviewed in [8]). The vital necessity of PR is indicated by the typical PR phenotype: mutants with defects in PR metabolism grow only in an atmosphere with elevated CO_2_ concentrations. When cultivated under ambient CO_2_ conditions (currently 0.041% CO_2_ in air) growth is impaired or even fully inhibited (reviewed in [9]). By the concerted action of nine enzymatic steps (Figure 1), the metabolic repair pathway leads to the detoxification of 2-PG and recycles 75% of the carbon contained in 2-PG to regenerate 3-PGA, which is resupplied to the Calvin–Benson (CB) cycle for the regeneration of the acceptor molecule ribulose 1,5-bisphosphate and production of triose phosphates. The remaining 25% are lost in the form of CO_2_ during the glycine decarboxylation reaction (reviewed in [8,10]). Reduction in photosynthetic efficiency and yield is estimated to reach about 20% of the CO_2_ previously fixed by photosynthesis in C_3_ plants under temperate climate conditions and can be even higher under hot and dry conditions [10]. Consequently, PR—“respiration in light”—is considered as a wasteful process. However, besides the detoxification of 2-PG, there are additional beneficial traits of the pathway: Firstly, since PR metabolism dissipates energy and reducing power its action lowers the potential of photoinhibition [11]. Secondly, PR metabolism is not an isolated pathway but is highly metabolically interconnected (reviewed in [12,13,14]). Besides the carbon recycling, it is also intertwined with nitrogen [15] and sulphur metabolism [16], and serves the production of amino acids (glycine, serine, glutamate, cysteine) and activated one-carbon units [10,17].

## 2. Metabolite Channeling in Photorespiratory Metabolism

In plants, the PR pathway is distributed over the three organelles chloroplast, peroxisome, and mitochondrion (Figure 1). The organellar compartmentalization allows embedding of the PR pathway into the signature metabolic alleys of the specific organelle. To enable efficient flux of metabolites through the pathway and also supply with co-factors for involved enzymes, specific transport steps are inevitable. In the following, the current knowledge about those transport processes at the plastidial, peroxisomal, and mitochondrial membranes is presented. A list of identified transport proteins involved in PR in *Arabidopsis thaliana* (henceforth Arabidopsis) is given in Table 1. 

### 2.1. Transport Processes at Chloroplasts

Chloroplasts, the starting and end point of the PR pathway, are surrounded by two membranes, namely the outer and the inner envelope. The outer envelope contains porins, which make it permeable to low molecular weight molecules. In contrast, the inner envelope forms a permeability barrier for metabolites and ions. As such, the shuttle across the inner envelope requires specific transport proteins [26,27]. Early studies on isolated chloroplasts from spinach and pea leaves showed that the core substrates of the PR pathway, glycerate and glycolate, are both shuttled across the chloroplast inner envelope in a carrier-mediated process. It was initially proposed that the metabolite shuttle was mediated by the same carrier protein in an anion-exchange reaction. Subsequent experiments also suggested a proton-driven symport as transport mode for glycolate [28,29,30,31,32]. It took about 40 years until the respective genes encoding for those transporters were identified and characterized on a molecular level in Arabidopsis [18,19]. In 2013, the plastidial glycolate/glycerate transporter PLGG1 was discovered by co-expression analysis. Knockout mutants deficient for PLGG1 show a typical PR phenotype and accumulate intermediates of the PR pathway, such as glycolate, glycine, and glycerate. Biochemical studies with recombinant PLGG1 protein as well as O_2_ flux analyses of wildtype and *plgg1* knockout plants eventually revealed its role in PR as a plastidial glycolate/glycerate translocator [18]. Four years later, yeast complementation assays as well as phenotypic analyses of a knockout mutant identified the bile acid sodium symporter 6 (BASS6) as a plastidial glycolate transporter in Arabidopsis [19]. To identify a transporter involved in PR, South and colleagues exploited that PR mutants are impaired in photosystem II activity and thus display reduced F_v_/F_m_ chlorophyll fluorescence in low CO_2_ conditions [19,33]. Similar to the *plgg1* mutant, the *bass6* mutant accumulates photorespiratory intermediates, such as glycolate and glycine but not glycerate, indicating its role as glycolate but not glycerate transporter. A double-knockout mutant of both *plgg1* and *bass6* contains significantly higher amounts of glycolate than either of the single-mutant lines or the wildtype. Interestingly, accumulation of serine and glycine reverts back to wildtype levels, indicating an overall alteration of PR metabolism [19]. Together, PLGG1 and BASS6 allow balanced export of two molecules of glycolate against one molecule of glycerate [18,19].

The first Arabidopsis PR transporter to be identified was a chloroplast dicarboxylate carrier in the early 1980s. The *dct* mutant was identified in a forward genetic approach developed by Chris and Shauna Sommerville in William Ogren’s laboratory. The mutant is lacking a transporter of the inner envelope, which is capable of catalyzing the flux of aspartate, 2-oxoglutarate (2-OG), malate, and glutamate [34,35]. Soon after, it was demonstrated that the transport of dicarboxylates across the chloroplast inner envelope requires at least two distinct carriers, later termed as 2-OG/malate translocator (DiT1/OMT) and glutamate/malate translocator (DiT2.1/DCT1) [36,37]. Both proteins exhibit overlapping substrate specificities. While both DiT1/OMT and DiT2.1/DCT1 are specific for dicarboxylates, such as malate, 2-OG, fumarate, and succinate, DiT2.1/DCT1 also accepts the amino acids glutamate and aspartate. Comprehensive genetic and biochemical studies supported the initial model of a two-translocator system for the refixation of ammonia in the chloroplast by the glutamine synthetase (GS)/ferredoxin-dependent glutamine:oxoglutarate (FdGOGAT) system. DiT1/OMT imports 2-OG into plastids in counter exchange with malate export. DiT2.1/DCT1 exports glutamate against cytosolic malate import, resulting in zero net-malate transport [20,21,38,39,40]. During PR, ammonia is released by the glycine decarboxylase (GDC) multi enzyme system in the mitochondria, and refixed by the GS/FdGOGAT system in the chloroplasts. As reviewed before, ammonium import into chloroplasts likely requires active transport or channeling [12]. However, such a transporter has not been identified to date. Thus, it remains elusive how and in which form ammonium crosses the plastid inner envelope.

### 2.2. Transport Processes at Peroxisomes

In the early 1970s, Tolbert’s laboratory at the Michigan State University discovered that peroxisomes in photosynthetic plant tissues play an essential role in PR. Tolbert and his colleagues intensively studied the peroxisomal enzymes involved in the fate of glycolate by labelling experiments, which is known as the glycolate or Tolbert pathway [41,42]. To functionally integrate leaf peroxisomes within the PR metabolism, a high flux of core intermediates across the peroxisomal membrane has to be coordinated [13]. For example, glycolate and serine have to enter peroxisomes, whereas glycerate and glycine have to be exported (Figure 1) for further conversion [14].

Beyond the transfer of core metabolites, PR-associated reactions within peroxisomes require the shuttling of additional molecules [14]. The reduction of hydroxypyruvate to glycerate by peroxisomal HPR1 depends on NADH, which is provided via the malate/oxaloacetate (OAA) shuttle [43]. This redox shuttle comprises two transport steps: the import of malate and the export of OAA. For the transamination of glyoxylate to glycine by glutamate:glyoxylate aminotransferase (GGAT), the amino group donor glutamate has to be transferred from chloroplasts to peroxisomes, where in return its 2-keto acid 2-OG has to be shuttled back into plastids to close the PR nitrogen cycle (Figure 1).

The uptake of tightly bound cofactors into peroxisomes for the PR enzymes, such as flavin mononucleotide (FMN)-containing GOX [44] or pyridoxal phosphate (PLP)-dependent aminotransferase [45,46], can be neglected. In contrast to chloroplasts and mitochondria, the peroxisomal matrix proteins can fold, acquire cofactors, and assemble into oligomers in the cytosol before targeting to peroxisomes [47]. Only for the coenzyme NAD^+^ [48] does a specific import mechanism exist at the peroxisomal membrane in plants (Figure 1).

Since the lipid bilayer functions as a main permeability barrier, most solutes cannot freely pass the peroxisomal membrane [13,49]. Channeling PR metabolites into and out of peroxisomes is achieved by integral membrane proteins that mediate the passage of a variety of PR intermediates. The recent model for peroxisomes suggests that the peroxisomal membrane contains two types of transporters: non-selective pore-forming diffusion channels and highly specific carrier proteins (for review, see [13,49]). Porins allow the transport of small hydrophilic solutes with molecular masses up to 300–400 Da, such as carboxylic acids and amino acids, whereas carrier-type transporters are involved for the transfer of larger molecules, such as NAD^+^ [13,49]. However, our current knowledge about the molecular identity of these transport proteins responsible for shuttling PR intermediates across the peroxisome membrane is rather limited.

First studies investigating the traffic of PR metabolites at the peroxisomal membrane were performed with leaf peroxisomes from spinach. The peroxisomal PR cycle was stimulated in vitro by adding glycolate, serine, glutamate, and malate to these intact organelles and analyzed by detecting the formation of glycerate [50,51,52]. These experiments indicated the existence of efficient uptake systems for these molecules across the peroxisomal membrane, allowing functional PR metabolism in isolated spinach peroxisomes.

Further electrophysiological lipid bilayer measurements using enriched peroxisomal membranes characterized a porin-like channel in spinach leaf peroxisomes [53,54]. This high-abundant channel is strongly selective for negatively charged solutes and displays a broad permeability for structurally diverse inorganic and organic anions. In respect to peroxisomal PR metabolism, this specific porin facilitates the transport of the anionic intermediates glycolate, glycerate, glutamate, malate, OAA, and 2-OG, but does not allow the passage of the zwitter-ionic amino acids glycine and serine (for review see [55]). Such a peroxisomal channel represents an efficient transport system allowing high flux of most of the metabolic intermediates through the PR cycle (Figure 1).

In Arabidopsis, the peroxisomal membrane protein of 22 kDa (PMP22) is proposed to be responsible for the pore-like channel activities, which has been described above [56]. This protein belongs to a small eukaryotic family of non-selective pore-forming channels, which are distributed to mitochondria and peroxisomes in human, mouse, yeast, and plants [13]. However, if the Arabidopsis PMP22 contributes to the permeability of PR, intermediates for the peroxisomal metabolism needs to be investigated in the future.

It is still an open question how the amino acids glycine and serine are shuttled through the peroxisomal membrane during PR. In plants, a high number of amino acid transporter classified into three major families are known. While numerous plasma membrane-localized amino acid carriers have been reported, only a few members have been found in plastids and mitochondria, but no member of this carrier group has been identified in peroxisomes so far [57].

To provide the peroxisomal malate dehydrogenase as part of the redox shuttle with NAD, Arabidopsis possesses a peroxisomal NAD carrier, called PXN [48,58]. This carrier protein imports NAD^+^ into peroxisomes to allow redox reactions in the peroxisomal lumen (Figure 1). In case of PR, NAD^+^ is reduced to NADH via the oxidation of malate to OAA, providing NADH for the HPR1 reaction. The Arabidopsis loss-of-function mutants for PXN do not display any obvious growth defects under PR conditions. Instead, dynamic light conditions, such as high-light fluctuations, triggered a significant photosynthetic defect in the *pxn* plants, whereas elevated CO_2_ levels completely rescued the observed phenotype [59]. It is assumed that the loss of PXN leads to an impairment of photosynthesis, because the PR pathway is disturbed due to an imbalance of reducing equivalents. Peroxisomal NAD^+^ imported by PXN might compensate for an increased NADH demand of the HPR1 reaction during PR under abiotic stress conditions [59].

### 2.3. Transport Processes at Mitochondria 

Similar to plastids, mitochondria are surrounded by two membranes, namely the outer and inner mitochondrial membrane. The first of these two membranes is permeable to molecules smaller than 5 kDa [60]. In contrast, metabolites rely on specific translocators in order to cross the inner mitochondrial membrane. Arabidopsis mitochondria contain at least 128 different transport proteins, many of which belong to the mitochondrial carrier family [61]. To date, none of them could be assigned to function either as glycine or as serine transporters. Earlier studies on isolated mitochondria from pea leaves suggested that the uptake of both core metabolites of the PR pathway, glycine and serine, is likely mediated by non-specific diffusion [62,63]. In contrast, a similar study on isolated mitochondria from spinach leaves proposed a concentration-dependent transport system. Glycine and serine are actively transported across the inner mitochondrial membrane at concentrations lower than 0.5 mM, whereas the diffusion process dominates at concentrations higher than 0.5 mM [64]. However, it remains elusive to date how glycine and serine cross the mitochondrial inner membrane and, if present, which transport proteins catalyze the putative shuttle of one or both metabolites.

During the combined reaction of GDC and serine hydroxymethyltransferase (SHMT) in the mitochondria, one molecule of serine is formed out of two molecules of glycine. Additionally, ammonia, NADH, and CO_2_ are released in equimolar amounts. It is assumed that ammonia diffuses towards the chloroplast where it is reassimilated by the GS/FdGOGAT system. However, given the toxicity of free ammonia, it was speculated that ammonia is transported towards the plastids in the form of an amino acid, such as glutamine or citrulline, utilizing an ornithine–citrulline or glutamate–glutamine shuttle system [65]. Both scenarios require the presence of a GS in mitochondria [65]. GS was previously hypothesized to be dual-localized to chloroplasts and mitochondria based on localization studies using fluorescent fusion-proteins [66]. However, in a recent proteomic study, it was reported that GS exists only in 26 copies per mitochondrion [67], which does not meet the demand for the high fluxes during PR, and is therefore negligible. In addition to a putative mitochondrial ammonia transporter, transporters of GDC and SHMT cofactors or their precursors remain to be identified. There are at least five transport steps necessary to provide GDC and SHMT with their respective cofactors (Vitamin B_6_ vitamers, lipoate or octanoate, malonate, folate precursor 6-hydroxymethyldihydropterine, NAD^+^). They were previously reviewed by Eisenhut and coworkers [68].

Recent advances have been made by identifying transporters of auxiliary metabolic pathways, such as the uncoupling proteins UCP1 and UCP2 in Arabidopsis [24,25]. UCP1 and UCP2 have a broad substrate spectrum. Next to the amino acids glutamate and aspartate, they also accept the dicarboxylates malate, succinate, and malonate to a lesser extent [25]. A recent study proposed that UCP1 and UCP2 contribute to the shuttle of redox equivalents during PR by catalyzing an electroneutral aspartate/glutamate exchange [25]. Aspartate and glutamate are substrate and product of the mitochondrial glutamate:OAA transaminase, which produces OAA that is required for NAD^+^-recycling by mitochondrial malate dehydrogenase. Analyses of Arabidopsis *ucp1* knockout mutants support this hypothesis. Even though *ucp1* mutants do not show a typical photorespiratory phenotype, they exhibit reduced levels of malate, reduced CO_2_ assimilation rates, and a strong reduction in glycine oxidation rate [24,25]. Double-knockout mutants of both *UCP1* and *UCP2* do not show any synergistic effect. Given the fact that UCP2 has been alternatively reported to reside in the membrane of the Golgi apparatus [69], its involvement in PR is still under debate [70,71]. In addition to UCP1, redox equivalents might be shuttled in the form of OAA/malate. The Arabidopsis genome contains three dicarboxylate carriers (DIC1-3), which are capable of efficiently transporting different dicarboxylates, such as malate, oxalate, malonate, succinate, and OAA [72]. However, their physiological role remains elusive as to date mutant studies are not available.

In 2013, the mitochondrial carrier protein À BOUT DE SOUFFLE (BOU) was identified in a co-expression analysis with PR genes in Arabidopsis [22]. Knockout mutants in *BOU* show a strong PR phenotype and accumulate intermediates of the PR pathway. The study proposed a transport function of BOU linked with the activity of GDC and SHMT. The transport substrate, however, remained enigmatic [22]. Recently, it was demonstrated that BOU facilitates mitochondrial glutamate transport in yeast [23]. Glutamate is essential for polyglutamylation of tetrahydrofolate (derivate of Vitamin B_9_), a cofactor of the T-protein of GDC and the SHMT. However, polyglutamylating enzymes are present in the chloroplasts and the cytosol as well, and it remains to be clarified how and which form of Vitamin B_9_ is transported across the mitochondrial membrane [73]. In a recent review, it was suggested that BOU might play a role in mitochondrial protein synthesis by supplying the mitochondria with glutamate [71]. Alternatively, glutamate might not be the only substrate of BOU. Besides glutamate, Porcelli and coworkers tested many additional substrates, such as aminoadipate, aspartate, asparagine, and glutamine. Transport activity could only be detected for L-homocysteine sulfinic acid, a substrate analog of glutamate [23]. However, the study lacks molecules that are related to PR, such as glycine, serine, and cofactors of the GDC/SHMT reaction. Further studies will be necessary to identify the physiological role of BOU with respect to PR.

## 3. Significance of Transport Steps in Synthetic Bypasses to PR

**Figure 2:**
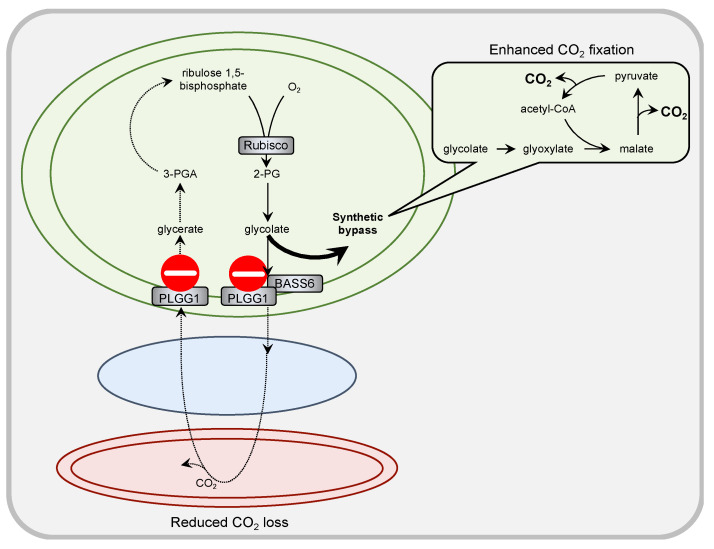
Significance of the plastidial transporter PLGG1 in anticipated flux distribution between native and synthetic route by South et al. [77] in PR metabolism. Silencing of the plastidial glycolate/glycerate transporter PLGG1 allows rerouting of major metabolite flux into the synthetic bypass. In this way, a large portion of the carbon contained in glycolate is released as CO_2_ in close proximity to Rubisco within the chloroplast, thus enhancing CO_2_ fixation. At the same time, CO_2_ loss is reduced since less glycolate continues the native PR route with subsequent mitochondrial decarboxylation reaction.

PR is frequently considered as a wasteful process: CO_2_ loss and energy consumption lower the photosynthetic efficiency and yield of plants, respectively. To counteract yield penalty, scientists developed different strategies. One tested strategy aimed at eliminating enzymatic bottlenecks, such as the mitochondrial glycine-to-serine conversion (reviewed in [14,74]). Another strategic concept envisages synthetic bypasses to PR that circumvent mitochondrial glycine decarboxylation and thus avoid release of CO_2_ and ammonia. In first attempts, glycolate-oxidizing pathways were implemented into Arabidopsis chloroplasts, either on the basis of bacterial [75] or plant [76] enzymes. An increase in biomass was observed for both engineering strategies under controlled growth conditions [75,76]. The so far most efficient bypass (Figure 2) was engineered by South and coworkers ([77], reviewed in [14,78]). They used tobacco as model crop and installed a mitochondrial glycolate dehydrogenase from the green alga *Chlamydomonas reinhardtii* and a peroxisomal malate synthase from *Cucurbita maxima* into chloroplasts. In the resulting engineered chloroplasts, Rubisco produced 2-PG is dephosphorylated by native 2-PG phosphatase. Glycolate is then oxidized by green algal glycolate dehydrogenase to yield glyoxylate, which, under control of transgenic malate synthase, reacts with acetyl-CoA to form malate. Malate is converted by the native chloroplast enzymes malic enzyme and pyruvate dehydrogenase into acetyl-CoA and two molecules of CO_2_. In total, the synthetic bypass facilitates the decomposition of glycolate into CO_2_ with regeneration of acetyl-CoA for renewed malate biosynthesis within the chloroplast only. The released CO_2_ elevates the CO_2_ to O_2_ ratio next to Rubisco and enhances the carboxylation versus the oxygenation activity of the enzyme. Furthermore, since mitochondrial ammonia release is also reduced, less energy needs to be spent for its reassimilation. As a consequence, transgene tobacco plants showed in greenhouse screens an increased biomass by 18% and in field trials by 10%. Strikingly, silencing of the chloroplast glycolate/glycerate transporter PLGG1 by RNAi technique and hence directing a large amount of glycolate into the bypass route even enhanced crop biomass by 24% compared to wild-type plants in both greenhouse and field trials [77]. PLGG1 works in tandem with BASS6 to ensure stoichiometric exchange of two molecules of glycolate against one molecule of glycerate at the chloroplast envelope [19]. Future studies will investigate whether additional silencing of BASS6 will result in even higher growth stimulation [77]. Due to the reduced but not fully abolished transcription of *plgg1*, in the transgene bypass plants, flux through the native PR pathway is likely not fully blocked but reduced. This allows continued generation of amino acids and one-carbon units, with simultaneous significant reduction in carbon loss. Though the actual numbers in flux distribution between native and synthetic route have not been determined yet, the results by South and colleagues [77] clearly demonstrate the significance and power of metabolite flux control by PR transport proteins.

In future attempts, advanced synthetic bypasses will be tested. Those systematically designed bypasses will combine existing and new-to-nature enzymes to ideally function in a carbon-conserving way and boost plant productivity [79]. Like the South bypass, those routes will also take place in the chloroplast only. Thus, the activities of PLGG1 and BASS6 will be a determinator for flux distribution through the native and the synthetic route.

## 4. Excursion: Metabolite Channeling in C_4_ Photosynthesis

Reduction of PR can increase carbon fixation and yield [80], and plants evolved mechanisms for reduction of PR by separating the Rubisco activity either temporally (crassulacean acid metabolism, CAM) or spatially (C_4_) from a primary CO_2_ fixation step by phosphoenolpyruvate carboxylase (PEPC). In this way, CAM plants possess remarkable water use efficiency [81]. C_4_ plants are characterized by high light, water, and nitrogen use efficiencies enabling crops species such as maize, sorghum, and miscanthus to achieve high biomass production and yield [82]. As demonstrated for a *GOX* mutant in maize, the presence of a working PR pathway is however essential for C_4_ species under ambient conditions [83].

In contrast to Rubisco, PEPC catalyzes just a carboxylation reaction. It is superior at CO_2_ fixation under conditions of low CO_2_ (e.g., when stomata are closed), high O_2_ or high temperatures. In the large majority of C_4_ species, the mesophyll cells are devoid of Rubisco, and the entering CO_2_ is fixed by PEPC producing a 4-carbon (C_4_) metabolite. The C_4_ metabolites malate and aspartate diffuse to the bundle sheath cell through plasmodesmata where they are decarboxylated again producing a high CO_2_ atmosphere around Rubisco. The establishment C_4_ photosynthesis is connected to major reconstruction of metabolic networks in the mesophyll and bundle sheath cells as well as anatomical adjustments. Additionally, operation of the C_4_ pathway also depends highly on inter- and intracellular transport activities. While C_3_ photosynthesis relies on one transporter for the formation of a three-carbon product, up to 30 transport proteins can be necessary for the synthesis via the NADP malic enzyme (NADP-ME) decarboxylation C_4_ subtype [84].

Already in C_3_ species, efficient trapping of photorespired CO_2_ can be achieved by changes to the bundle sheath shape and size, its organelles number, size, and arrangement [85]. In a next decisive evolutionary step, the PR decarboxylation reaction moves completely from the mesophyll to the bundle sheath, realized by cell specific expression of the glycine decarboxylate P-protein (GLDP) [86,87]. Consequently, glycine accumulates in the mesophyll cells and moves to the bundle sheath cells, where high decarboxylation activity increases the CO_2_ concentration around the bundle sheath cell Rubisco. Plants with such a glycine shuttle mechanism can be found in many phylogenetic groups and can be identified by significantly reduced CO_2_ compensation points [85,88]. The operation of the pathway goes along with increased transport activities. The continuous transport of glycine from mesophyll to bundle sheath requires rebalancing of the C, N, and redox metabolism between the two cell types [89,90,91]. So far, experimental evidence for the identity of the involved balancing compounds is missing. Modeling approaches identified glutamate and 2-OG, aspartate and malate, or alanine and pyruvate as possible shuttled metabolites between the mesophyll and bundle sheath cell [89]. Depending on the nature of the compound, additional intracellular transporter activities could be involved, including carriers from the PR pathway for the shuttling of glutamate and 2-OG between peroxisome, cytosol, and plastid or the shuttling of malate between plastid and cytosol. Significant changes in transcript abundance of the dicarboxylate transporters could, however, not be found when the closely related species *Moricandia arvensis* or *M. suffruticosa* with a glycine shuttle and *M. moricandioides* without a glycine shuttle were compared [88].

**Figure 3:**
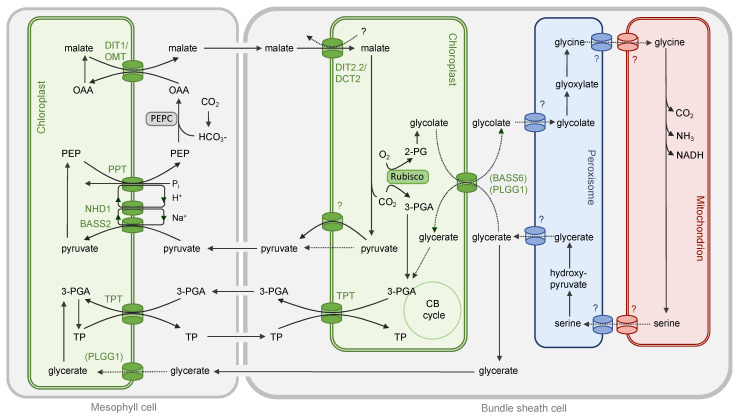
Transport steps in the NADP-ME subtype of C_4_ photosynthesis including CB cycle and PR. The minor C_4_ aspartate shuttle has been omitted. The CO_2_-fixing enzymes in the two cell types are shown in boxes, and the names of the transporters are shown in the color of the associated organelle. The transporters responsible for cell specific exchange of PR metabolites have not been confirmed experimentally and are shown in brackets. Metabolite transport mechanisms without experimental evidence are shown in dashed lines.

Further general reduction of PR is possible when, on top of the glycine shuttle, PEPC activity rises in the mesophyll cells [85,89]. The processes described in the following paragraphs all relate to the operation of the NADP-ME subtype of C_4_ photosynthesis. CO_2_ entering the cells would partly be converted into bicarbonate by the carbonic anhydrase and fixed by PEPC. The produced OAA would be converted into malate in the plastids of the mesophyll cells—requiring additional dicarboxylate transporters for intracellular exchange (Figure 3). In *Flaveria* species displaying different stages between C_3_ and C_4_ photosynthesis, the increasing activity of PEPC is associated with increased transcript abundance of the dicarboxylate transporters DiT1/OMT and DiT2.2/DCT2 [89]. In the C_4_ species, *F. trinervia* and *F. bidentis*, all genes connected to the PR pathway were transcriptionally downregulated; this also includes genes related to N assimilation. The only exceptions were genes for the dicarboxylate transporters DiT1/OMT and DiT2.2/DCT2 indicating their involvement in the C_4_ shuttle pathway [92,93]. In maize leaves, DiT1/OMT and DCT1 were more abundant in the mesophyll cell fraction, while DCT2 and DCT3 accumulated in the bundle sheath [94]. Besides malate and 2-OG, Dit1/OMT proteins also possess high affinity for OAA, suggesting that this transporter is capable of OAA and malate exchange at the mesophyll chloroplast membrane during C_4_ photosynthesis [20,21,95] (Figure 3). Mesophyll specific upregulation of the DiT1/OMT transporter can also be found in other species using NADP-ME for decarboxylation such as *Sorghum bicolor* [94], and *S. viridis* [96]. Experiments overexpressing Dit1/OMT in rice resulted in strong reduction of photosynthesis and growth, and it could only be rescued by concomitant expression of DiT2.2/DCT2. The results suggest that the DiT1/OMT transporter activity needs to be regulated in close coordination with photosynthesis [97]. The DiT1/OMT gene was also shown to be under strong evolutionary pressure during evolution of C_4_ in NADP-ME type grasses [98].

In the bundle sheath cells of NADP-ME species, malate again needs to enter the chloroplast in exchange with pyruvate (Figure 3). The high abundance of *DiT2.2/DCT2* copies in bundle sheath cells of NADP-ME species suggest that the corresponding transporters are involved in the C_4_ related metabolite exchange. In maize, DCT2 seems to enable transport of malate into the BS plastid [99]. The transport mechanism of DCT2 in the bundle sheath is, however, unclear. The malate transport could be coordinated with aspartate transport, but, if malate would be exchanged with aspartate, a second transporter for aspartate uptake into the plastid would be necessary [99]. In *S. bicolor*, another member of the gene family, DCT4, seems to be the predominant transporter in the bundle sheath [100]. For the export of pyruvate out of the bundle sheath plastid, no transporter could be identified so far. It has been suggested that diffusion of pyruvate in its electroneutral form could be possible [101].

Completing the C_4_ cycle, pyruvate diffuses back to the mesophyll cell for regeneration of PEP. The step is catalyzed by the plastid localized PPDK and therefore requires exchange of pyruvate and PEP at the mesophyll chloroplast membrane. In dicotyledonous C_4_ species from the genus *Flaveria*, the process is mediated by the BASS2/NHD/PPT transport system. BASS2 transports pyruvate in a sodium dependent manner, and the sodium influx is balanced by a sodium–proton antiporter (NHD1) [102]. In maize and related C_4_ species, a different, not sodium dependent pyruvate transport system seems to be active [103]. The proton is transported back across the plastidial membrane together with PEP and in exchange with phosphate by the PPT. In contrast to the dicarboxylate transporters described above, PPT was not present in high abundance in the C_3_ leaf and had to obtain its C_4_ related expression pattern during C_4_ evolution [104].

The C_4_ transport systems differ between the C_4_ subtypes [105,106,107]. A comparably strong transcript abundance of the dicarboxylate transporters DiT1/OMT and DiT2.2/DCT2 was not detected in the leaves of NAD-ME *Cleome* species [108] and *Panicum virgatum* [105]. In this subtype, mitochondrial dicarboxylate carriers (DIC) were enhanced [105]. Nevertheless, the BASS2/NDH/PPT transport system described for NADP-ME *Flaveria* species was also found in an NAD-ME species from the Cleomaceae [102].

In C_4_ species, the CB and PR cycles start with Rubisco activity in the bundle sheath. However, the activity of the light reactions and consequently reductive power is higher in the mesophyll cells, especially in the NADP-ME subtype. The reduction steps of the CB cycle from 3-PGA to triose phosphate are therefore localized in the mesophyll. This requires additional metabolite shuttling between the cells and transport of 3-PGA and triose phosphate across plastidial membranes in both cell compartments [109]. In C_4_ species, the triose phosphate translocator (TPT) is generally transcribed at high levels [105]. In the last step of PR, glycerate kinase catalyzes the production of 3-PGA. In coordination with the CB cycle, this step is also localized in the mesophyll cells. It requires the transport of PR glycerate from bundle sheath to the mesophyll. The PLGG1 translocator exchanges glycolate and glycerate at the plastid membrane [18], but, in the C_4_ leaf, the transport of both metabolites would be largely uncoupled. In the bundle sheath, BASS6 could contribute to glycolate export [19], but, in the mesophyll plastid, the absence of PR glycolate would limit glycerate uptake by the PLGG1 translocator.

In general, efficient C_4_ photosynthesis relies heavily on the presence and activity of numerous transporters and intercellular diffusion processes. The facilitation of high metabolite fluxes remains one of the challenges for the engineering of the C_4_ cycle into C_3_ species [107,110].

## 5. Conclusions and Perspectives

Transport proteins connect the cellular compartments involved in PR metabolism and thus enable efficient metabolite flow. Apparently, the molecular identification of genes encoding those proteins proves challenging. Future attempts may include additional screening strategies, such as the chlorophyll fluorescence-based assay used for the identification of BASS6 [19]. Alternatively, higher order mutants and physiological analyses of biochemically characterized transporters (e.g., DICs in mitochondria) might identify transporters that are involved in PR but do not show the classical PR phenotype due to overlapping substrate specificities and functional redundancy. Next to forward genetic approaches and co-expression analyses [111], those approaches might serve as powerful tools to identify the enigmatic transport proteins involved in PR.

## Figures and Tables

**Table 1:** List of identified PR transporters in *Arabidopsis thaliana.*

Transporter	Abbreviation	Substrate	*Arabidopsis thaliana* Identifier	References
plastidial glycolate/glycerate transporter	PLGG1	glycerate/glycolate	At1g32080	[18]
bile acid sodium symporter 6	BASS6	glycolate	At4g22840	[19]
2-OG/malate translocator	DiT1/OMT1	2-OG/malate	At5g12860	[20,21]
glutamate/malate translocator	DiT2.1/DCT1	glutamate/malate	At5g64290	
À BOUT DE SOUFFLE	BOU	glutamate	At5g46800	[22,23]
uncoupling protein 1	UCP1	aspartate/glutamate	At3g54110	[24,25]

## Data Availability

Not applicable.
